# Changes in the Epidemiology of Influenza and Respiratory Syncytial Virus During 2020–2022 Relative to the Pre‐COVID‐19 Pandemic Period (2017–2020) From Systematic Sentinel Syndromic Surveillance in South Africa

**DOI:** 10.1111/irv.70207

**Published:** 2026-01-11

**Authors:** Sibongile Walaza, Jocelyn Moyes, Anne von Gottberg, Nicole Wolter, Amelia Buys, Fahima Moosa, Mignon du Plessis, Gary Reubenson, Jeremy Nel, Heather J. Zar, Halima Dawood, Ebrahim Variava, Mvuyo Makhasi, Omphile Mekgoe, Fathima Nabby, Neydis Baute, Jackie Kleynhans, Susan Meiring, Vanessa Quan, Cheryl Cohen

**Affiliations:** ^1^ Centre for Respiratory Diseases and Meningitis National Institute for Communicable Diseases of the National Health Laboratory Service Johannesburg South Africa; ^2^ School of Public Health, Faculty of Health Sciences University of the Witwatersrand Johannesburg South Africa; ^3^ School of Pathology, Faculty of Health Sciences University of the Witwatersrand Johannesburg South Africa; ^4^ Division of Medical Microbiology, Department of Pathology, Faculty of Health Sciences University of Cape Town Cape Town South Africa; ^5^ Department of Paediatrics & Child Health, Rahima Moosa Mother & Child Hospital, Faculty of Health Sciences, School of Clinical Medicine University of the Witwatersrand Johannesburg South Africa; ^6^ Department of Medicine, Faculty of Health Sciences University of the Witwatersrand Johannesburg South Africa; ^7^ Department of Paediatrics and Child Health, Red Cross War Memorial Children's Hospital, and SA‐MRC Unit on Child & Adolescent Health University of Cape Town Cape Town South Africa; ^8^ Department of Medicine Greys Hospital Pietermaritzburg South Africa; ^9^ Caprisa University of KwaZulu‐Natal Pietermaritzburg South Africa; ^10^ Department of Paediatrics Klerksdorp‐Tshepong Hospital Complex Klerksdorp South Africa; ^11^ Department of Medicine Klerksdorp‐Tshepong Hospital Complex Klerksdorp South Africa; ^12^ Department of Health KwaZulu‐Natal, Pietermaritzburg Metropolitan Hospitals University of KwaZulu‐Natal Pietermaritzburg South Africa; ^13^ Department of Paediatrics Mapulaneng Hospital Hazyview South Africa; ^14^ Divison of Public Health Surveillance and Response National Institute for Communicable Diseases of the National Health Laboratory Service Johannesburg South Africa

**Keywords:** nonpharmaceutical interventions, respiratory infection, SARS‐CoV‐2, seasonality, surveillance

## Abstract

**Background:**

Nonpharmaceutical interventions, implemented during the COVID‐19 pandemic, affected the transmission of other respiratory pathogens.

**Methods:**

Systematically collected respiratory illness surveillance data and consistent case definitions were used to describe changes in influenza and respiratory syncytial virus (RSV)–associated outpatient visits and hospitalisations in South Africa during the first 3 years of the COVID‐19 pandemic relative to a pre‐COVID‐19 pandemic period (2017–2019).

**Results:**

In 2020, influenza circulation almost ceased. In 2021 an out‐of‐season circulation was observed with a return to prepandemic timing, albeit with a higher peak in 2022. During the pandemic period, influenza‐associated influenza‐like illness (ILI) was more common in those aged ≥ 5 years compared to < 6 months. Patients with influenza‐associated severe respiratory illness (SRI) were less likely to be ≥ 45 years versus < 6 months and less likely to be admitted to ICU (aOR 0.2, 95% CI 0.04–0.8).

RSV circulation declined at the start of the pandemic, with an out‐of‐season spring resurgence in 2020 followed by a return to prepandemic timing in 2021 and a higher peak in 2022. During the pandemic, compared to the prepandemic period, patients with RSV‐associated SRI were more likely to be aged 1–4 years (aOR 1.5, 95% CI 1.2–1.8) versus < 6 months and less likely to be admitted to ICU (aOR 0.5, 95% CI 0.3–0.8).

**Conclusion:**

We report low levels of influenza circulation and out‐of‐season RSV circulation in 2020 with changes in the age distribution of cases and risk of ICU admission. Return to prepandemic timing was earlier for RSV, with higher seasonal peaks for influenza‐associated ILI and RSV‐associated SRI in 2022.

## 1 Background

Seasonal epidemics of influenza and respiratory syncytial virus (RSV) cause a significant burden of disease among children and adults annually [1, 2, 3, 4, 5, 6]. Prior to the severe acute respiratory syndrome virus 2 (SARS‐CoV‐2) pandemic, the RSV and influenza seasons in South Africa occurred in autumn and winter, respectively [7, 8]. Like SARS‐CoV‐2, influenza and RSV are primarily transmitted through respiratory droplets, aerosols and indirect contact with contaminated surfaces.

South Africa implemented nonpharmaceutical interventions (NPIs) against SARS‐CoV‐2 in March 2020, varying over time from lockdown (stay at home except for workers in essential services), wearing of facemasks, physical distancing and promotion of improved hygiene, such as handwashing. All of these have the potential to disrupt transmission of other respiratory pathogens. Data from several studies, mostly including data from the first year of the pandemic, suggested that the NPIs resulted in an overall decrease in the transmission of respiratory viruses [9, 10, 11, 12].

During the first few months of the pandemic, we reported a decrease in the circulation of influenza and RSV in South Africa compared to previous years [10]. However, the period included in the analysis did not provide the opportunity to assess if these changes were sustained, as the restrictions were gradually lifted, or whether there was a rebound increase in cases and a possible increase in severity of illness following a period of low transmission. In addition, changes in the age distribution and severity of cases were not evaluated. These data will be important for anticipating possible effects of widespread implementation of NPIs in future pandemics. Some countries have reported an increase in RSV‐associated admissions after the pandemic, with some attributing the increase to changes in testing rather than a surge in transmission, whereas others attributed the increase to the easing of nonpharmaceutical interventions [13, 14].

Using systematically collected respiratory illness surveillance data, we describe changes in the epidemiology of influenza and RSV among children and adults in South Africa during the first 3 years of the COVID‐19 pandemic compared to the preceding 3 years.

## 2 Methods

### 2.1 Study Design and Population

#### 2.1.1 Syndromic Surveillance Programmes

We used data from two previously described syndromic surveillance programmes in South Africa, namely, the influenza‐like illness (ILI) programme at primary health clinics in four provinces and the severe respiratory illness (SRI) programme at hospitals in five provinces [8, 15, 16]. All potentially eligible patients from Monday to Friday were screened, and patients meeting the surveillance case definitions were approached for consent to participate.

#### 2.1.2 Case Definitions for Patient Enrolment

ILI: Outpatient with either temperature ≥ 38°C or history of fever and cough for a duration of ≤ 10 days [17].

SRI: For hospitalised cases, we expanded the WHO SARI case definition, which requires fever and cough and symptom onset within 10 days to include any patient hospitalised for severe respiratory illness regardless of symptom duration and included any physician diagnosed LRTI (including pleural effusion and sepsis in children). SRI was defined as a hospitalised person with symptoms of any duration meeting age‐specific clinical inclusion criteria on admission:

- Children aged 2 days to < 3 months: physician‐diagnosed suspected sepsis or lower respiratory tract illness (LRTI) irrespective of signs and symptoms.
- Children aged 3 months to < 5 years: physician‐diagnosed LRTI, including bronchitis, bronchiolitis, pneumonia and pleural effusion (with or without fever).
- Individuals aged ≥ 5 years: LRTI with fever (≥ 38°C) or history of fever and cough.

### 2.2 Sample Collection and Laboratory Testing Procedures

Respiratory samples (combined nasopharyngeal [NP] and oropharyngeal [OP] swabs during 2017–2019 and NP or miturbinate swabs during 2020–2022) were collected in universal transport medium on the day of consultation for ILI or within 48 h of admission for SRI. Samples were stored at 4°C–8°C at the surveillance site laboratory until transported in cooler boxes with ice to the National Institute for Communicable Diseases (NICD) for processing and testing within 72 h of collection. From 2017 to February 2021, nasopharyngeal/nasal swabs were tested for Influenza A, Influenza B and RSV using a commercial multiplex real‐time reverse transcriptase PCR assay (Fast‐Track Diagnostics, Luxembourg). From 1 March 2021, samples were tested for Influenza A, Influenza B and RSV using the Allplex SARS‐CoV‐2/Flu A/Flu B/RSV kit (Seegene, Seoul, South Korea). A specimen was considered positive for any of the detected pathogens if the respective targets were detected with cycle threshold (Ct) values ≤ 40 according to manufacturer instructions. Influenza A and B and RSV positive samples were further subtyped using the CDC influenza and RSV subtyping kits, respectively, available through the International Reagent Resource (IRR).

### 2.3 Data Collection

Surveillance officers collected demographic data, medical history, clinical presentation, clinical management and in‐hospital outcome data through structured interviews and by record review. Initially, surveillance data were collected using paper‐based forms and transitioned to web‐based data capture during the COVID‐19 pandemic. The data were checked for completeness and validity.

### 2.4 Analysis

We defined the prepandemic period as January 2017 through February 2020 and the pandemic period as March 2020 through December 2022 for the multivariable analysis.

We compared the following between the 2017–2019 and 2020–2022 periods, among individuals enrolled in ILI and SRI, separately:

- Weekly detection rate for influenza and RSV.
- Mean annual detection rate for influenza and RSV by age group.

We then compared the demographic and clinical characteristics of influenza‐ or RSV‐associated ILI and SRI cases between the prepandemic (January 2017 to February 2020) and the pandemic (March 2020 to December 2022) periods, using multivariable logistic regression. To account for clustering by site, random effects were applied in logistic regression models. Variables with *p* < 0.10 on univariate analysis were evaluated for inclusion in the multivariable models. Nonsignificant variables at *p* ≥ 0.05 were dropped using manual backward elimination. Stata Version 18 (StataCorp Limited, College Station, TX) was used for analysis.

## 3 Results

### 3.1 Characteristics of Patients With ILI and SRI during the Pandemic and Prepandemic Period

From 2017 through 2022, a total of 7910 patients with ILI were enrolled, 3953 (50%) and 3957 (50%) during the prepandemic and pandemic period, respectively (Table S1). During the same period, 26,101 hospitalised patients with SRI were enrolled, 14,781 (57%) and 11,320 (43%) during the prepandemic and pandemic periods, respectively (Table S2). The median age of enrolled patients with ILI was 11 years (interquartile range IQR 2–36 years) and was older during the pandemic period (26 years, IQR 5–40 vs. 5 years, IQR 1–28, *p* < 0.01). For SRI cases, the median age was 1 year (IQR 0–34 years) and older for cases enrolled during the pandemic (2 years, IQR 0–36 vs. 1 year, IQR 0–32, *p* < 0.001).

In the pandemic, compared to the prepandemic period, the percentage of SRI patients with diabetes increased from 2% (297/15,294) to 5% (482/10,764), *p* < 0.001, and the percentage of patients living with HIV (LHIV) decreased from 24% (3591/15,098) to 17% (1686/9959), *p* < 0.001. The percentage of SRI patients receiving oxygen therapy increased from 44% (6786/15,281) to 68% (7365/10,784), *p* < 0.001. Overall mortality was 4% (977/26,001), higher during the pandemic (5%, 522/10,728) compared to the prepandemic (3%, 455/15,273, *p* < 0.001) period (Table S2).

### 3.2 Influenza Detection and Circulation

In patients with ILI, the influenza detection rate was 12% (461/3935) during the prepandemic period, decreasing to 9% (373/3954) in the pandemic period (*p* < 0.001). The influenza detection rate was 2% (23/1285) in 2020, 9% (146/1566) in 2021 and 18% (204/1135) in 2022.

In patients with SRI, the influenza detection rate was 6% (814/14,936) during the prepandemic period decreasing to 4% (413/10,686) during the pandemic period (*p* < 0.001). The influenza detection rate was 1% (33/3307) in 2020, 5% (71/3697) in 2021 and 5% (215/4201) in 2022.

**FIGURE 1:**
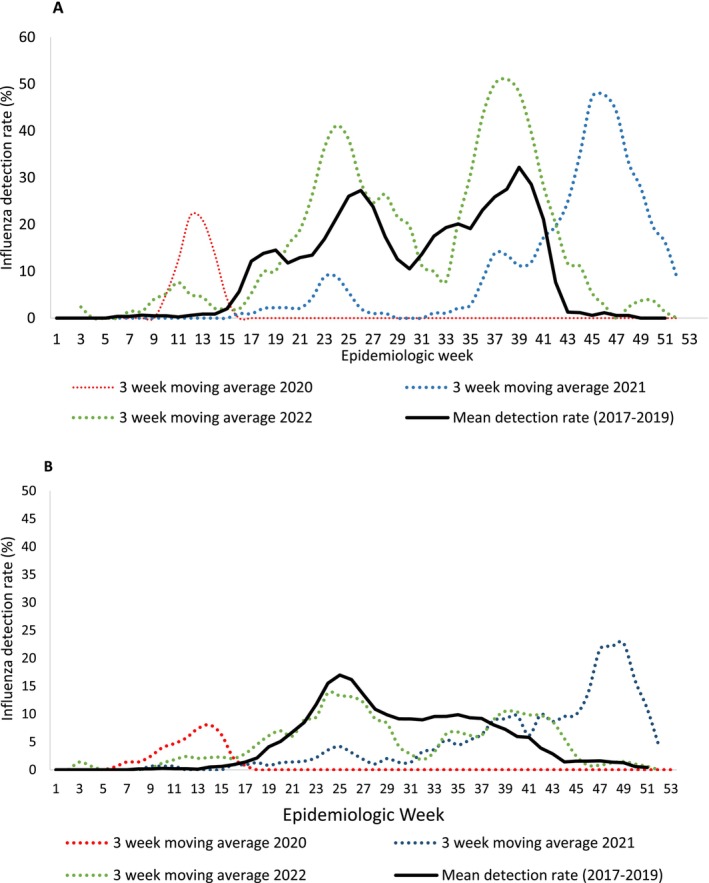
Influenza detection rate and mean detection rate (2017–2019) among patients attending outpatient clinics with influenza like illness (A) and hospitalised with severe respiratory illness (B), 2017–2022.

In both ILI and SRI, from 2017 through 2019, influenza circulation was biphasic with a peak in Week 25 and a second peak in Weeks 32–39 (Figure 1A,B). In 2020, influenza circulated prior to Week 16 but was then not detected until 2021 when there was an increase in circulation in the second half of the year with an unusual spring peak in Weeks 43–51. In 2022, the timing of influenza circulation was similar to the prepandemic period, albeit with a higher peak in ILI.

**FIGURE 2:**
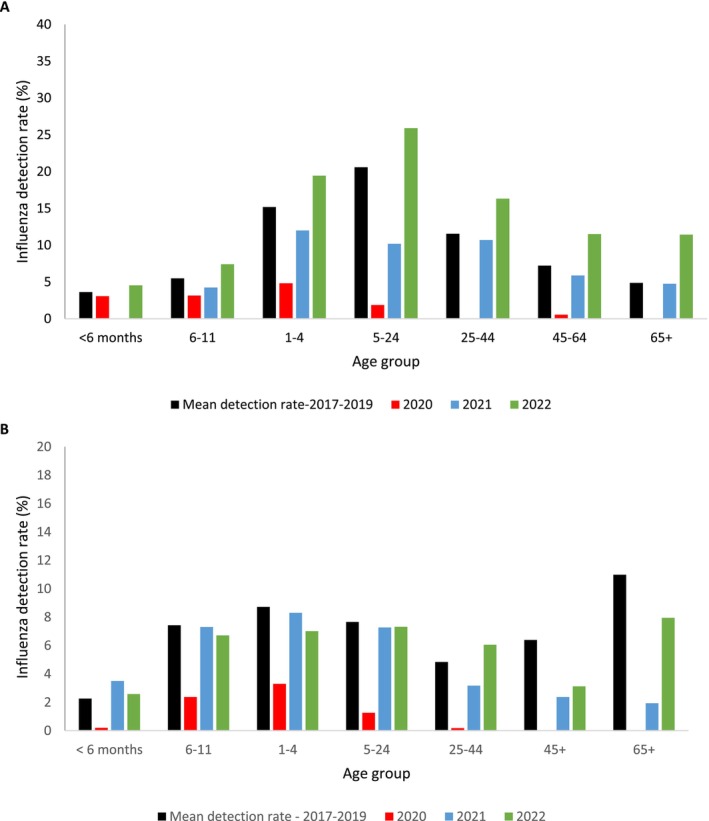
Detection rate of laboratory‐confirmed influenza by age group and year, among patients attending outpatient clinics with influenza like illness (A) and hospitalised with severe respiratory illness (B), 2017–2022. The detection of each age group was compared between mean detection rate of 2017–2019 and 2020–2022.

During the prepandemic period, the influenza detection rate among ILI cases increased by age group, peaking in the 5‐ to 24‐year age group (Figure 2A). In 2020, the detection rates were significantly lower than in the prepandemic period for all age groups ≥ 1 year. In 2021, the detection rate was lower than the prepandemic period in the 5‐ to 24‐year age group (10% [95% CI 7.0–14.0] vs. 20% [95% CI 17.1–23.1]). In 2022, detection rates were similar to the prepandemic period (Figure 2A).

Among SRI cases, in 2017–2019, the influenza mean detection rates were higher in young children (< 5 years) and in older individuals (> 65 years) (Figure 2B). Compared to the mean detection rate in 2017–2019, in 2020, influenza detections were significantly lower for all age groups. In 2022, the detection rates remained significantly lower in the 45‐ to 64‐year (2%, 95% CI 1.3–3.9 vs. 6%, 95% CI 5.2–7.7) and ≥ 65‐year age groups (2%, 95% CI 0.7–4.1 vs. 10%, 95% CI 8.8–13.1) compared to the mean detection rate for 2017–2019.

### 3.3 RSV Detection and Circulation

In patients with ILI, the RSV detection rate was 7% (263/3935) during the prepandemic period, decreasing to 5% (186/3954) during the pandemic years, *p* < 0.001. RSV detection rate was 6% (81/1285) in 2020, 3% (54/1556) in 2021 and 5% (51/1135) in 2022, respectively.

In patients with SRI, the RSV detection rate was 16% (2424/14956) during 2017–2019 and 16% (1659/10686) during 2020–2022 (*p* = 0.141). RSV detection rate was 16% (543/3307) in 2020, 10% (383/3697) in 2021 and 18% (747/4201) in 2022.

**FIGURE 3:**
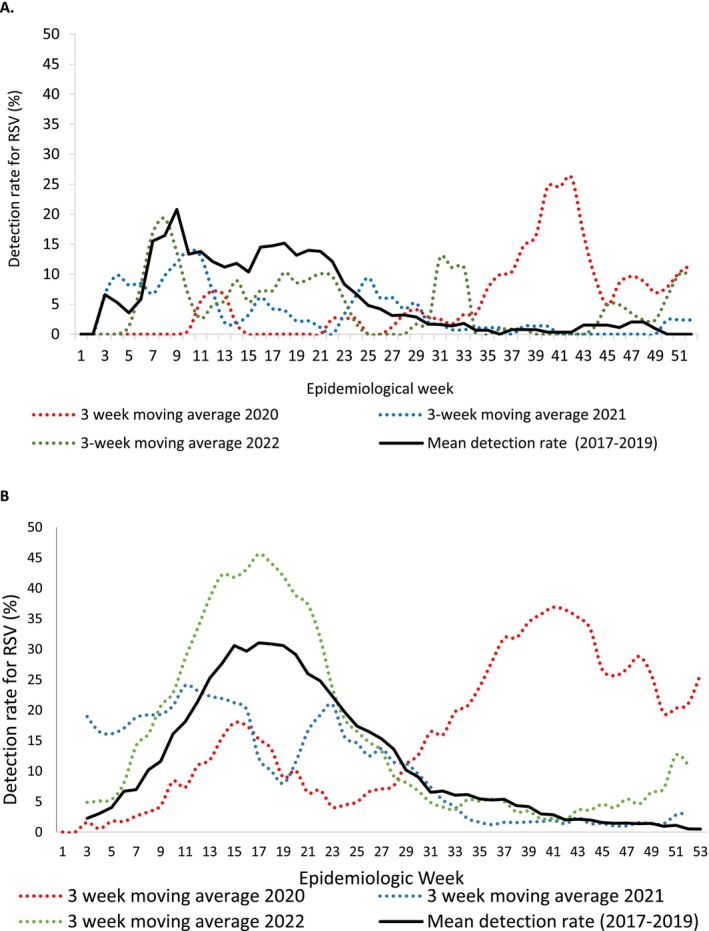
Respiratory syncytial virus (RSV) detection rate and mean detection rate (2017–2019) among patients attending outpatient clinics with influenza like illness (A) and hospitalised with severe respiratory illness (B), 2017–2022.

In both ILI and SRI, from 2017 through 2019, RSV circulated in the first half of the year, peaking in Weeks 15–17 in SRI (Figure 3A,B). In 2020, RSV circulated at a lower level in the first part of the year, peaking in Week 15, and then decreased, with a second higher peak in Week 41. In 2021, RSV circulated in the first half of the year at lower detection rates than usual, followed by a higher than usual detection rate in 2022.

**FIGURE 4:**
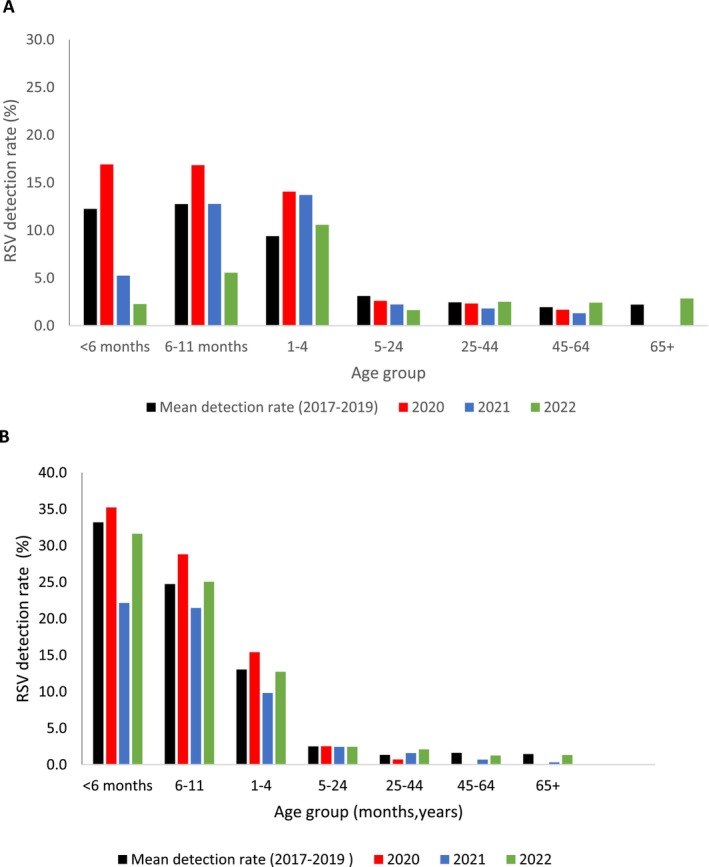
Detection rate of laboratory‐confirmed respiratory syncytial virus by age group among patients attending outpatient clinics with influenza‐like illness (A) and hospitalised with severe respiratory illness (B), 2017–2022.

During the 2017–2019 period, the average RSV detection rate among ILI cases was highest in children aged 6–11 months and decreased with increasing age (Figure 4A). The detection rates by age group among ILI cases did not change significantly in 2020–2022. Among SRI cases, during the 2017–2019 period, the RSV detection rates were highest in children aged < 6 months and decreased with increasing age (Figure 4B). Compared to the mean detection rate for the 2017–2019 period, in 2021, the detection rate was significantly lower among children aged < 6 months (23%, 95% CI 19.6–24.8 vs. 33%, 95% CI 31.8–34.5).

### 3.4 Influenza‐Associated Outpatient Illness and Hospitalisation in the Prepandemic (January 2017 to February 2019) and Pandemic (March 2020 to December 2022) Period

There were 461 patients with influenza‐associated ILI in the prepandemic period and 373 in the pandemic period (Table 1A). The median age of influenza‐associated ILI patients was higher during the pandemic than during the prepandemic period (11 years, IQR: 4–33 vs. 6 years, IQR: 3–24, *p* < 0.001). On multivariable analysis, adjusting for site, in the pandemic period compared to the prepandemic period, patients with influenza‐associated ILI were more likely to be aged ≥ 5 years (5–24 years [aOR 4.7, 95% CI 1.4–15.7], 25–44 years [aOR 13.3, 95% CI 3.9–45.3], 45–64 years [aOR 7.5, 95% CI 2.1–27.0] and ≥ 65 years [aOR 9.4, 95% CI 1.5–57.8]) as compared to < 6 months, of other race (aOR 1.9, 95% CI 1.0–3.5) and to present with symptoms of < 5 days duration (aOR 1.5, 95% CI 1.1–2.2) (Table 1A).

**TABLE 1A:** Characteristics of patients with influenza‐like illness (ILI) testing positive for influenza in the pre‐COVID‐19 pandemic (January 2017 to February 2022) and pandemic (March 2020 to December 2022) periods.

Characteristic	Prepandemic *N* = 461	Pandemic *N* = 373	OR (95% CI)	aOR (95% CI)
Age in years, median (IQR)	7 (3–24)	11 (4–33)		
Age group				
< 6 months	15/460 (3)	4/373 (1)	Reference	Reference
6–11 months	18/460 (4)	9/373 (2)	1.9 (0 5–7.3)	2.3 (0.5–10.0)
1–4 years	145/460 (32)	90/373 (24)	2.3 (0.7–7.2)	2.9 (0.9–9.6)
5–24 years	171/460 (37)	116/373 (31)	2.5 (0.8–8.0)	4.7 (1.4–15.7)
25–44 years	75/460 (16)	110/373 (30)	5.5 (1.7–17.2)	13.3 (3.9–45.3)
45–64 years	32/460 (7)	38/373 (10)	4.4 (1.3–14.8)	7.5 (2.1–27.0)
≥ 65 years	4/460 (0.8)	6/373 (2)	5.6 (1.1–30.1)	9.4 (1.5–57.8)
Sex				
Male	221/461 (46)	198/373 (53)	1.3 (1.0–1.8)	1.4 (1.0–1.8)
Female	250/461 (54)	175/373 (47)	Reference	Reference
Race				
Other	53/461 (12)	138/372 (37)	4.5 (3.2–6.5)	1.9 (1.1–3.5)
Black	408/461 (89)	234/372 (63)	Reference	Reference
Province				
KwaZulu‐Natal	226/461 (49)	87/373 (23)	Reference	Reference
Western Cape	78/461 (17)	166/373 (45)	5.5 (3.8–8.0)	5.2 (2.9–9.5)
North West	157/461 (34)	120/373 (32)	2.0 (1.4–2.8)	1.9 (1.3–2.8)
Asthma				
No	448/461 (97)	358/371 (97)	Reference	
Yes	13/461 (3)	13/371 (4)	1.3 (0.6–2.7)	
Diabetes				
No	456/461 (99)	367/371 (99)	Reference	
Yes	5/461 (1)	4/371 (1)	1.0 (0.3–3.7)	
Living with HIV				
No	389/451 (86)	323/362 (89)	Reference	
Yes	62/451 (14)	39/362 (11)	3.7 (0.5–1.2)	
Symptom duration				
0–4 days	334/461 (72)	297/372 (80)	1.5 (1.1–2.1)	1.5 (1.1–2.2)
≥ 5 days	127/461 (28)	75/372 (20)	Reference	Reference

Abbreviations: aOR, adjusted odds ratio; CI, confidence interval; OR, odds ratio.

In the pandemic period compared to the prepandemic period, patients with influenza‐associated SRI were less likely to be in older age groups (45–64 years [aOR 0.4, 95% CI 0.2–0.7] and ≥ 65 years [aOR 0.4, 95% CI 0.2–0.7] vs. < 6 months) and to be admitted to ICU (aOR 0.2, 95% CI 0.04–0.9). They were more likely to present with symptoms < 5 days duration (aOR 1.5, 95% CI 1.2–2.0) and to receive oxygen (aOR 2.5, 95% CI 1.9–3.3) (Table 1B).

**TABLE 1B:** Characteristics of patients with severe respiratory illness testing positive for influenza in the pre‐COVID‐19 pandemic (January 2017 to February 2022) and pandemic (March 2020 to December 2022) periods.

Characteristic	Prepandemic*N* = 814	Pandemic *N* = 413	OR (95% CI)	aOR (95% CI)
Age in years, median (IQR)	3 (1–36)	2 (17)		
Age group				
< 6 months	105/814 (13)	73/413 (18)	Reference	Reference
6–11 months	129/814/814 (16)	65/413 (16)	0.7 (0.5–1.1)	0.8 (0.5–1.1)
1–4 years	243/814 (30)	159/413 (39)	0.9 (0.6–1.3)	1.0 (0.7–1.4)
5–24 years	61/814 (8)	29/413 ()	0.7 (0.4–1.2)	0.9 (0.5–1.6)
25–44 years	120/814 (15)	45/413 (11)	0.5 (0.3–0.8)	0.7 (0.4–1.1)
45–64 years	96/814 (12)	24/413 (6)	0.4 (0.2–0.6)	0.4 (0.2–0.7)
≥ 65 years	60/814 (7)	18/413 (4)	0.4 (0.2–0.8)	0.4 (0.3–0.8)
Sex				
Male	408/814 (50)	220/413 (53)	Reference	
Female	406/814 (50)	193/413 (47)	0.9 (0.7–1.1)	
Race				
Other	172/814 (21)	72/412 (18)	Reference	
Black	642/814 (79)	340/412 (83)	1.2 (0.9–1.6)	
Province				
Gauteng	172/814 (21)	89/413 (22)	1.6 (1.1–2.5)	1.33 (0.8–2.1)
KwaZulu‐Natal	117/814 (14)	60/413 (15)	1.6 (1.0–2.6)	1.6 (1.0–2.6)
Mpumalanga	100/814 (12)	50/413 (12)	1.6 (1.0–2.6)	2.3 (1.4–3.9)
Western Cape	295/814 (36)	173/413 (42)	1.9 (1.2–2.8)	1.6 (1.0–2.5)
North West	130/814 (16)	41/413 (10)	Reference	Reference
Asthma				
No	776/814 (95)	396/408 (98)	Reference	
Yes	38/814 (5)	12/408 (3)	0.6 (0.3–1.2)	
Diabetes				
No	790/814 (97)	11/408 (97)	Reference	
Yes	24/814 (3)	11/408 (3)	0.9 (0.4–1.9)	
Living with HIV				
No	625/803 (78)	321/374 (86)	Reference	
Yes	178/803 (22)	53/374 (14)	0.6 (0.4–0.8)	
Symptom duration				
0–4 days	421/788 (53)	270/413 (65)	1.6 (1.3–2.1)	1.5 (1.2–2.0)
≥ 5 days	367/788 (47)	143/413 (35)	Reference	
Hospital duration				
< 4 days	353/814 (43)	274/412 (45)	0.95 (0.7–1.2)	
≥ 4 days	461/814 (57)	145/412 (55)	Reference	
Oxygen therapy				
No	447/814 (55)	152/412 (37)	Reference	
Yes	367/814 (45)	260/412 (63)	2.1 (1.6–2.6)	2.5 (1.9–3.3)
ICU admission				
No	798/814 (98)	410/412 (99)	Reference	Reference
Yes	16/814 (2)	2/412 (1)	0.2 (0.1–1.1)	0.2 (0.0–0.9)
Outcome				
Discharged	794/813 (98)	402/412 (98)	Reference	
Died	19/813 (2)	10/412 (2)	1.0 (0.5–2.3)	

Abbreviations: aOR, adjusted odds ratio; CI, confidence interval; OR, odds ratio.

### 3.5 RSV‐Associated Outpatient Illness and Hospitalisation During the Prepandemic and Pandemic Period

The median age of RSV‐associated ILI patients was higher during the pandemic than during the prepandemic period (3 years, IQR 1–8 years, vs. 2 years, IQR 1–4 years, *p* < 0.001) (Table 2A). In the pandemic compared to the prepandemic period, RSV‐associated ILI cases were more likely to be in the older age groups (1–4 years [aOR 2.4, 95% CI 1.2–5.0], 5–24 years [aOR 2.7, 95% CI 1.1–6.8], 25–44 years [aOR 19.4, 95% CI 6.8–54.9] and 45–64 years [aOR 10.0, 95% CI 2.8–35.2] vs. < 6 months), be of other races versus black (aOR 2.0, 95% CI 1.1–3.9) and to present with symptoms < 5 days duration versus ≥ 5 days (aOR 3.1, 95% CI 1.7–5.8).

**TABLE 2A:** Characteristics of patients with influenza‐like illness testing positive for respiratory syncytial virus in the pre‐COVID‐19 pandemic (January 2017 to February 2022) and pandemic (March 2020 to December 2022) periods.

Characteristic	Prepandemic *n* = 263	Pandemic *n* = 186	OR, 95% CI	AOR, 95% CI
Age in years, median (IQR)	2 (1–4)	3 (1–8)		
Age group				
< 6 months	50/263 (19)	13/186 (7)	Reference	Reference
6–11 months	47/263 (18)	25/186 (13)	2.1 (0.9–4.5)	1.7 (0.7–3.99)
1–4 years	115/263 (44)	90/186 (48)	3.0 (1.5–5.9)	2.4 (1.2–5.0)
5–24 years	27/263 (10)	19/186 (10)	2.7 (1.2–6.3)	2.7 (1.1–6.8)
25–44 years	14/263 (5)	27/186 (15)	7.4 (3.1–18.0)	19.4 (6.8–54.9)
45–64 years	7/263 (3)	11/186 (6)	6.0 (2.0–18.7)	10.0 (2.8–35.2)
≥ 65 years	3/263 (1)	1/186 (1)	1.3 (0.1–13.4)	1.1 (0.1–13.14)
Sex				
Male	132/263 (50)	98/186 (53)	Reference	
Female	131/263 (50)	88/186 (47)	0.9 (0.6–1.3)	
Race				
Other	71/263 (27)	102/186 (55)	3.3 (2.2–4.9)	2.1 (1.1–3.9)
Black	192/263 (73)	84/186 (45)	Reference	Reference
Province				
KwaZulu‐Natal	68/263 (26)	42/186 (23)	2.1 (1.2–3.7)	2.6 (1.3–5.2)
Western Cape	100/263 (38)	116/186 (62)	3.9 (2.4–6.5)	4.0 (1.8–9.0)
North West	95/263 (36)	28/186 (15)	Reference	Reference
Asthma				
No	260/263 (99)	184/186 (99)	Reference	
Yes	3/263 (1)	2/186 (1)	0.9 (0.2–5.8)	
Diabetes				
No	262/263 (100)	186/186 (100)	Reference	
Yes	1/263 (0.4)	0/186 (0)	Not determined	
Living with HIV				
No	249/260 (96)	165/173 (95)	Reference	
Yes	11/260 (5)	8/173 (5)	1.1 (0.4–2.8)	
Symptom duration				
0–4 days	188/263 (71)	165/185 (89)	3.3 (1.9–5.6)	3.1 (1.7–5.8)
≥ 5 days	75/263 (29)	20/185 (11)	Reference	

Abbreviations: aOR, adjusted odds ratio; CI, confidence interval; OR, odds ratio.

The median age of RSV‐associated SRI patients was similar during the prepandemic and pandemic periods (0 years, IQR 0–1, vs. 0, IQR 0–1, *p* = 0.10) (Table 2B). In the pandemic compared to the prepandemic period, patients with RSV‐associated SRI were more likely to be aged 1–4 years (aOR 1.5, 95% CI 1.2–1.8) versus < 6 months, to be of Black race versus other (aOR 1.2, 95% CI 1.0–1.4), to present with symptom duration of < 5 vs. ≥ 5 days (aOR 1.6; 95% CI 1.4–1.9) and to receive oxygen therapy (aOR 3.2, 95% CI 2.8–3.7). They were less likely to be admitted to ICU (aOR 0.5, 95% CI 0.3–0.8). There was no significant difference in mortality (aOR 1.3, 95% CI 0.6–2.7).

**TABLE 2B:** Characteristics of patients with severe respiratory illness testing positive for respiratory syncytial virus in the pre‐COVID‐19 pandemic (January 2017 to February 2022) and pandemic (March 2020 to December 2022) periods.

Characteristic	Prepandemic*n* = 2424	Pandemic *n* = 1659	OR, 95% CI	AOR, 95% CI
Age in years, median (IQR)	0 (0–1)	0 (0–1)		
Age group				
< 6 months	1549/2424 (64)	1017/1659 (61)	Reference	Reference
6–11 months	424/2424 (17)	286/1659 (17)	1.0 (0.9–1.2)	1.1 (0.9–1.4)
1–4 years	365/2424 (15)	310/1659 (19)	1.3 (1.1–1.5)	1.5 (1.2–1.8)
5–24 years	20/2424 (1)	13/1659 (1)	1.0 (0.5–2.0)	1.7 (0.8–3.5)
25–44 years	33/2424 (1)	22/1659 (1)	1.0 (0.6–1.8)	1.4 (0.8–2.6)
45–64 years	25/2424 (1)	8/1659 (1)	0.5 (0.2–1.1)	0.5 (0.2–1.2)
≥ 65 years	8/2424 (0.3)	3/1659 (0.2)	0.6 (0.2–2.2)	0.8 (0.2–3.2)
Male sex				
Male	1347/2424 (56)	879/1659 (53)	Reference	
Female	1077/2424 (44)	780/1659 (47)	1.1 (0.98–1.26)	
Race				
Other	750/2423 (31)	462/1657 (28)	Reference	Reference
Black	1673/2423 (69)	1195/1657 (72)	1.2 (1.0–1.3)	1.2 (1.04–1.4)
Province				
Mpumalanga	147/2424 (6)	75/1659 (4.5)	Reference	Reference
KwaZulu‐Natal	272/2424 (11)	139/1659 (8)	1.0 (0.7–1.4)	0.67 (0.5–1.0)
Gauteng	483/2424 (20)	358/1659 (22)	1.5 (1.1–1.9)	0.93 (0.7–1.3)
Western Cape	1367/2424 (56)	969/1659 (58)	1.4 (1.0–1.9)	1.2 (0.8–1.6)
North West	155/2424 (6)	118/1659 (7)	1.5 (1.0–2.2)	1.1 (0.8–1.6)
Asthma				
No	2404/2423 (99)	1633/1652 (99)	Reference	
Yes	19/2423 (1)	19/1652 (1)	1.5 (0.8–2.8)	
Diabetes				
No	2420/2424 (100)	1651/1652 (100)	Reference	
Yes	4/2424 (0)	1/1652 (0)	0.4 (0.0–3.3)	
Living with HIV				
No	2321/2401 (97)	1494/1529 (98)	Reference	
Yes	80/2401 (3)	35/1529 (2)	0.7 (0.5–1.0)	
Symptom duration				
0–4 days	1739/2412 (72)	1344/1659 (81)	1.65 (1.5–1.9)	1.58 (1.3–1.9)
≥ 5 days	673/2412 (28)	315/1659 (19)	Reference	Reference
Hospital duration				
< 4 days	1194/2423 (49)	754/1649 (46)	Reference	Reference
≥ 4 days	1229/2423 (51)	895/1649 (54)	1.2 (1.0–1.3)	1.23 (1.1–1.5)
Oxygen therapy				
No	1074/2423 (44.3)	352/1657 (21)	Reference	Reference
Yes	1349/2423 (55.7)	1305/1657 (79)	2.9 (2.6–3.4)	3.2 (2.8–3.7)
ICU admission				
No	2366/2422 (98)	1633/1657 (99)	Reference	Reference
Yes	56/2422 (2)	24/1657 (1)	0.6 (0.4–1.0)	0.5 (0.3–0.8)
In‐hospital outcome				
Discharged	2406/2422 (99)	1635/1649 (99)	Reference	
Died	16/2422 (1)	14/1649 (1)	1.3 (0.6–2.7)	

Abbreviations: aOR, adjusted odds ratio; CI, confidence interval; OR, odds ratio.

## 4 Discussion

Using the same case definition and systematically collected data from syndromic surveillance 3 years before and the first 3 years of the COVID‐19 pandemic, we report a decrease in influenza‐associated ILI and SRI in the first year of the pandemic, followed by an out of season increase in 2021 and a return to prepandemic trends, albeit a higher seasonal peak in ILI in 2022. Following the interruption of the typical South African RSV season in March 2020, we showed an out‐of‐season increase in RSV detections in the spring of the first year of the pandemic, which continued into 2021 followed by a return to prepandemic seasonal trends with a higher seasonal peak in 2022. The age distribution of influenza and RSV cases in both ILI and SRI varied between the two periods. During the pandemic, a higher proportion of patients with influenza‐associated ILI were in older age groups, whereas the reverse was true for influenza‐associated SRI. Children hospitalised with RSV‐associated disease during the pandemic period were more likely to be aged 1–4 years versus < 6 months compared to the prepandemic period. There was no significant difference in mortality during the two periods; however, the point estimates for influenza‐ and RSV‐associated mortality were higher.

Similar to other countries, we saw a decrease in influenza circulation during the first year of the COVID‐19 pandemic compared to the prepandemic years [9, 11, 18, 19, 20]. The resurgence in influenza circulation in 2021 was out of season; other countries reported activity lower than the prepandemic seasons or only reported sporadic influenza cases during this period [11, 21]. In South Africa, the peak detections during the first out of season resurgence in 2021 were similar to prepandemic seasons. During the third year of the pandemic, 2022, similar to other countries, influenza transmission followed similar trends to prepandemic seasons in South Africa, albeit at a higher seasonal peak [22]. Australia reported an early season in 2022 with numbers of influenza cases higher than in the 3 years before the pandemic [21].

The first restrictions, implemented in response to SARS‐CoV‐2, in March 2020 in South Africa, interrupted RSV transmission soon after the start of the RSV season. However, unlike influenza circulation, this interruption in circulation was brief; RSV cases increased out of season with the first easing of restrictions after a restrictive lockdown in 2020. Other countries reported similar interruptions in RSV circulation during the first year of the pandemic, with resurgence of RSV circulation reported mostly during the second year of the pandemic [23, 24, 25, 26]. Similar to other countries, the timing of the first resurgence of RSV was not in keeping with prepandemic seasons [23, 27, 28]. The United States of America, France and Australia reported interseasonal increases during spring/summer of 2021 versus the usual autumn/winter season [23, 24, 25, 27, 28].

During the resurgence of RSV and influenza in the pandemic years, the percentage of influenza hospitalisations was higher in young children, peaking at age 1–4 years, than in older age groups, and RSV‐associated SRI in children shifted to older childhood age groups. Similar to our study, other studies also reported an increase in RSV‐associated hospitalizations in older children or an increase in the median age of RSV‐associated illness during the pandemic years [26, 29, 30]. This could be explained by increased hospitalisations in RSV naïve older children who had missing RSV exposure in the first year of life due to the public health containment measures instituted at the beginning of the pandemic. A contrasting study in a tertiary care facility in Italy reported the majority of RSV‐associated hospitalisations during the resurgence in 2021 to be children aged < 12 months; investigators hypothesised that this could be due to young children with severe illness being prioritised for admission [31].

There was a lower ICU admission rate for both influenza and RSV cases during the pandemic; this is more likely due to the load in the hospital and ICUs, with ICU's beds being reserved for severe COVID and other more severe patients.

Our study did not show a significant increase in mortality in influenza‐ and RSV‐associated hospitalisations during the pandemic, although point estimates for mortality were higher for both. A study from the United States of America also did not report an increase in RSV‐associated severe illness compared to the prepandemic period [25]. Similarly, a study in New York reported no changes in influenza‐associated outcomes during the COVID‐19 pandemic compared to the prepandemic period [32]. This is different to some studies that reported a higher proportion of severe influenza‐ and RSV‐associated cases hospitalised during the pandemic period compared to the prepandemic period [25, 29, 30]. The increase in mortality reported in some studies may be due to an immunity gap created following a period without virus circulation leading to increased population vulnerability or hospitals being overwhelmed, especially during the first 2 years of the pandemic.

### 4.1 Strengths and Limitations

A strength of our study was the use of consistent surveillance methods, sites and case definition for case ascertainment before and during the pandemic. This allowed us to show the temporal changes in influenza and RSV including a longer period after the relaxation of restrictions implemented in response to COVID‐19 than other studies. Our data included both mild and severe influenza‐ and RSV‐associated illness. Our surveillance requires consent; therefore, we may have missed severe cases who were not able to give consent or children whose parents were not present; as a result, we may have underestimated the mortality. For this analysis, we excluded cases who only met the expanded case definition that was applied during the pandemic period in order to ensure consistency when comparing the two periods. Cases meeting the expanded COVID‐19 case definition may have differed in clinical presentation and outcome. In addition, our clinical case definition in children aged < 5 years includes pneumonia and pleural effusion. We acknowledge that pleural effusion, although described [33], would be extremely rare in cases of influenza and RSV infection, but our case definition is intentionally broad, aiming to capture both bacterial and viral cases of SRI. The fact that the case definitions remained constant in the prepandemic and pandemic periods suggests that the inclusion of pleural effusion on the case definition is unlikely to have introduced substantial bias for our main comparisons. For the multivariable analysis, we combined 2020–2022 cases as pandemic period; this may have limited our ability to tease out the year‐to‐year differences in the epidemiology of influenza and RSV. During the first year of pandemic, surveillance may have missed enrolling some cases due to surveillance staff absenteeism due to COVID‐19. Lastly, reporting incidence for influenza and RSV‐associated illness would have provided better assessment of changes in the epidemiology of these two pathogens but was not possible due to unavailable denominators.

In conclusion, COVID‐19 altered influenza‐ and RSV‐associated seasonal transmission and hospitalisation patterns. We report an unprecedented decrease in influenza transmission and hospitalisations during the first year of the pandemic followed by an out‐of‐season increase in cases in the second year and return, albeit with a higher peak, to the prepandemic trends in 2022. On the contrary, although the RSV season was interrupted at the start of the pandemic, we report an earlier, out‐of‐season resurgence of RSV during the first year of the pandemic followed by the return to prepandemic trends in 2021 and a higher peak in 2022. These results highlight the importance of continued systematic surveillance during the pandemic and suggest that nonpharmaceutical measures implemented for COVID‐19 had an impact on the trends of RSV and influenza and should be encouraged during influenza and RSV epidemics. This study also highlights the importance of conducting year‐round surveillance for these pathogens and providing real‐time data that could be used for public health action and response.

## Author Contributions

All authors approved the final manuscript as submitted and agree to be accountable for all aspects of the work. *Conceptualization*: Cheryl Cohen, Sibongile Walaza, Anne von Gottberg, Jocelyn Moyes and Nicole Wolter. *Data curation*: Sibongile Walaza, Mvuyo Makhasi and Fahima Moosa. *Formal analysis*: Sibongile Walaza. *Funding acquisition*: Cheryl Cohen and Sibongile Walaza. *Investigation and methodology*: Sibongile Walaza, Anne von Gottberg, Jocelyn Moyes, Fahima Moosa, Mignon du Plessis and Cheryl Cohen. *Writing* (*original draft preparation*): Sibongile Walaza. *Writing* (*review and editing*): Sibongile Walaza, Anne von Gottberg, Jocelyn Moyes, Nicole Wolter, Fahima Moosa, Amelia Buys, Gary Reubenson, Jeremy Nel, Susan Meiring, Vanessa Quan, Mvuyo Makhasi, Heather J. Zar, Halima Dawood, Jackie Kleynhans, Ebrahim Variava and Cheryl Cohen.

## Funding

The study was funded by the Centers for Disease Control and Prevention Foundation (NU51IP000930, U01IP001048 and 5U01IP001048); funds through the CDC under the terms of a subcontract with the African Field Epidemiology Network (AFENET) (AF‐NICD‐001/2021); and the Africa Centers for Disease Control and Prevention through a subaward from the Bill and Melinda Gates Foundation Grant Number INV‐018978, as well as the National Institute for Communicable Diseases, a division of the National Health Laboratory Service, South Africa. The funding agencies had no role in the development of the study protocol, data collection, analysis and interpretation, writing of the report or decision to submit.

## Ethics Statement

Both the ILI and SRI protocols were approved by the University of the Witwatersrand Human Research Ethics Committee (HREC), reference M180832 and M140824, respectively. Additional approvals were received from other HRECs. Written consent was obtained from participants aged ≥ 18 years and from parents/guardians of participants aged < 18 years. In addition to consent from parents/guardians, assent was obtained from participants aged 7–17 years.

## Conflicts of Interest

S.M. received an investigational grant from Sanofi Pasteur unrelated to this research. N.W. and A.v.G. received funding from Sanofi and the Gates Foundation. C.C. has received funding from the Wellcome Trust, US CDC, PATH, South African Medical Research Council, Sanofi and Gates Foundation unrelated to this work. S.W. has received funding from US CDC related to this work and Gates Foundation and Task Force for Global Health unrelated to this work. M.d.P. has received funding from the Gates Foundation and the National Research Foundation, South Africa, unrelated to this work. J.M. has received grant funds from Pfizer unrelated to this work.

## Supporting information


**Table S1:** Demographic description and clinical presentation of influenza‐like illness cases enrolled in the pre‐pandemic (Jan 2017‐Feb 2020) and pandemic (March 2020‐ Dec 2022) periods.
**Table S2:** Demographic description and clinical presentation of severe respiratory illness (SRI) cases enrolled in the pre‐pandemic (Jan 2017‐Feb 2020) and pandemic (March 2020‐ Dec 2022) periods.

## Data Availability

The data generated and analysed during this study contain potentially identifiable information and therefore have restricted access due to privacy and ethical issues. Access to aggregated data can be obtained by request to the corresponding author, Sibongile Walaza (sibongilew@nicd.ac.za), and will be subject to proof of an IRB‐approved protocol and signature of a data sharing agreement. Responses to requests will be within 4 weeks from request receipt.
