# Supplementary material for: Changes in the Epidemiology of Influenza and Respiratory Syncytial Virus During 2020–2022 Relative to the Pre‐COVID‐19 Pandemic Period (2017–2020) From Systematic Sentinel Syndromic Surveillance in South Africa
Source: Influenza Other Respir Viruses. 2026 Jan 11;20(1):e70207. doi: 10.1111/irv.70207 (PMC12790951; doi:10.1111/irv.70207)
Supplement: Supplementary file 1 — Table S1: Demographic description and clinical presentation of influenza‐like illness cases enrolled in the pre‐pandemic (Jan 2017‐Feb 2020) and pandemic (March 2020‐ Dec 2022) periods. Table S2: Demographic description and clinical presentation of severe respiratory illness (SRI) cases enrolled in the pre‐pandemic (Jan 2017‐Feb 2020) and pandemic (March 2020‐ Dec 2022) periods. [file IRV-20-e70207-s001.docx]

Changes in the epidemiology of influenza and respiratory syncytial virus during 2020-2022 relative to the pre-COVID-19 pandemic period (2017-2020) from systematic sentinel syndromic surveillance in South Africa

**Authors:**

Authors: Sibongile Walaza^1,2^, Jocelyn Moyes^1,2^ , Anne von Gottberg^1, 3,12^, Nicole Wolter^1,3^; Amelia Buys^1^, Fahima Moosa^1,3^, Mignon du Plessis^1,3^, Gary Reubenson^4^, Jeremy Nel^5^, Heather J Zar^6^, Halima Dawood^7,8^, Ebrahim Variava ^5,9^, Mvuyo Makhasi^1,2^,Omphile Mekgoe^10^, Fathima Nabby^13^, Neydis Baute^14^, Jackie Kleynhans^1,2^, Susan Meiring^11^, Vanessa Quan^11^, Cheryl Cohen^1,2^

Affiliation

^1^Centre for Respiratory Diseases and Meningitis, National Institute for Communicable Diseases of the National Health Laboratory Service, Johannesburg, South Africa

^2^School of Public Health, Faculty of Health Sciences, University of the Witwatersrand, Johannesburg, South Africa

^3^School of Pathology, Faculty of Health Sciences, University of the Witwatersrand, Johannesburg, South Africa

^4^Department of Paediatrics & Child Health, Rahima Moosa Mother & Child Hospital,Faculty of Health Sciences, School of Clinical Medicine, University of the Witwatersrand, , Johannesburg South Africa

^5^Department of Medicine, Faculty of Health Sciences, University of the Witwatersrand, Johannesburg, South Africa

^6^ Department of Paediatrics and Child Health, Red Cross War Memorial Children’s Hospital, and SA-MRC Unit on Child & Adolescent Health, University of Cape Town, Cape Town, South Africa.

^7^Department of Medicine, Greys Hospital, Pietermaritzburg, South Africa

^8^Caprisa, University of KwaZulu - Natal, Pietermaritzburg, South Africa

^9^ Department of Paediatrics, Klerksdorp-Tshepong Hospital Complex, Klerksdorp, South Africa

^10^Department of Medicine, Klerksdorp-Tshepong Hospital Complex, Klerksdorp, South Africa

^11^Divison of Public Health Surveillance and Response, National Institute for Communicable Diseases of the National Health Laboratory Service, Johannesburg, South Africa

^12^Division of Medical Microbiology, Department of Pathology, Faculty of Health Sciences, University of Cape Town, Cape Town

^13^Department of Health KwaZulu-Natal, Pietermaritzburg Metropolitan Hospitals, University of KwaZulu-Natal, Pietermaritzburg, South Africa

^14^Department of Paediatrics, Mapulaneng hospital, Hazy View, South Africa

**Supplementary Table 1A: Demographic description and clinical presentation of influenza-like illness cases enrolled in the pre-pandemic (Jan 2017-Feb 2020) and pandemic (March 2020- Dec 2022) periods**

| Characteristic | Total  n=7910 | Pre-pandemic period  n=3 953 (50) | Pandemic period  n=3 957 (50) | p-value |
| --- | --- | --- | --- | --- |
| Age in years, median (IQR) | 11 (2-36) | 5 (1-29) | 26 (5-41) |  |
| Age group |  |  |  | 0.001 |
| <6 months | 551/7 896 (6) | 424/3 939 (11) | 127/3 957 (3) |  |
| 6 -11 months | 600/7 896 (8) | 410/3 939 (10) | 190/3 957 (5) |  |
| 1-4 years | 1 852/7 896 (23) | 1 143/3 939 (29) | 709/3 957 (18) |  |
| 5-24 years | 1 733/7 896 (22) | 852/3 939 (22) | 881/3 957 (22) |  |
| 25-44 years | 1 906/7 896 (24) | 621/3 939 (16) | 1 285/3 957 (32) |  |
| 45-64 years | 1 038/7 896 (13) | 391/3 939 (10) | 647/3 957 (16) |  |
| ≥65 years | 216/7 896 (3) | 98/3 939 (2) | 118/3 957 (3) |  |
| Sex |  |  |  | 0.002 |
| Male | 3 718/7 908 (47) | 1 790/3 952 (45) | 1 928/3 956 (49) |  |
| Female | 4 190/7 908 (53) | 2 162/3 952 (55) | 2 028/3 956 (51) |  |
| Race |  |  |  | <0.001 |
| Other | 2 473/7 900 (31) | 800/3 949 (20) | 1 673/3 951 (42) |  |
| Black | 5 427/7 900 (69 | 3 149/3 949 (80) | 2 278/3 951 (58) |  |
| Province |  |  |  | <0.01 |
| KwaZulu-Natal | 2 177/7 910 (28 | 1 415/3 953 (36) | 762/3 957 (19) |  |
| Western Cape | 2 933/7 910 (37) | 1 053/3 953 (27) | 1 880/3 957 (47) |  |
| North West | 2 800/7 910 (35) | 1 485/3 953 (38) | 1 315/3 957 (33) |  |
| Asthma |  |  |  | 0.495 |
| No | 7 671/7 899 (97) | 3 843/3 952 (97) | 3 828/3 947 (97) |  |
| Yes | 228/7 899 (3) | 109/3 952 (3) | 119/3 947 (3) |  |
| Diabetes |  |  |  | 0.127 |
| No | 7 772/7 899 (98) | 3 897/3 952 (99) | 3 875/3 947 (98) |  |
| Yes | 127/7 899 (2) | 55/3 952 (1 | 72/3 947 (2) |  |
| Living with HIV |  |  |  | 0.079 |
| No | 6 675/7 648 (87) | 3 318/3 831 (87) | 3 357/3 817 (88) |  |
| Yes | 973/7 648 (13) | 513/3 831 (13) | 460/3 817 (12). |  |
| Symptom duration |  |  |  | 0.001 |
| 0-4 days | 5 538/7 906 (70) | 2 617/3 951 (66) | 2 921 (74) |  |
| ≥5 days | 2 368/7 906 (30) | 1 334/3 951 (34) | 1 034 (26) |  |

**Supplementary Table 1B: Demographic description and clinical presentation of severe respiratory illness (SRI) cases enrolled in the pre-pandemic (Jan 2017-Feb 2020) and pandemic (March 2020- Dec 2022) periods**

| Characteristic | Total  n=26 101 | Pre-pandemic period  n=15 302 (59%) | Pandemic period  n=10 799 (41%) | p-value |
| --- | --- | --- | --- | --- |
| Age in years, median (IQR) | 1 (0-34) | 1 (0-33) | 2 (0-36) |  |
| Age group |  |  |  | <0.001 |
| <6 months | 8 241/26 080 (32) | 4,945/15 281 (32) | 3 296/10 799 (31) |  |
| 6 -11 months | 3 027/26 080 (12) | 1 905/15 281 (13 | 1 122/10 799 (10) |  |
| 1-4 years | 5 309/26 080 (20) | 2 859/15 281 (19) | 2 450/10 799(23) |  |
| 5-24 years | 1 314/26 080 (5) | 815/15 281 (5) | 499/10 799 (5) |  |
| 25-44 years | 4 042/26 080 (16) | 2 562/15 281 (17) | 1 480/10 799 (14) |  |
| 45-64 years | 2 890/26 080 (11) | 1 594/15 281 (10) | 1 296/10 799 (12) |  |
| ≥65 years | 1 257/26 080 (5) | 601/15 281 (4 | 656/10 799 (6) |  |
| Sex |  |  |  | 0.827 |
| Female | 12 049/26 086 (46) | 7 056/15 295 (46) | 4 993/10 791 (46) |  |
| Male | 14 037/26 086 (54 | 8 239/15 295 (54) | 5 798/10 791 (54) |  |
| Race |  |  |  | <0.001 |
| Other | 6 234/26 078 (24) | 3 517/15 292 (23) | 2 717/10 786 (25) |  |
| Black | 19 844/26 078 (76) | 11 775/15 292 (77) | 8 069/10 786 (75) |  |
| Province |  |  |  | <0.001 |
| Gauteng | 5 737/26 101 (22) | 3 546/15 302 (23) | 2 191/10 799 (20) |  |
| KwaZulu-Natal | 3 924/26 101 (15) | 2 452/15 302 (16) | 1 472/ 10 799 (14) |  |
| Mpumalanga | 2 192/26 101 (8) | 1 178/15 302 (8) | 1 014/10 799 (9) |  |
| Western Cape | 10 940/26 101 (42) | 5 890/15 302 (39) | 5 050/10 799 (47) |  |
| North West | 3 308/26 101/26 101 (13) | 2 236/15 302 (15) | 1 072/10 799 (10) | 0.152 |
| Asthma |  |  |  |  |
| No | 25 284/26 057 (97) | 14 820/15 293 (97) | 10 464/10.764 (97) |  |
| Yes | 773/26 057 (3) | 473/15 293 (3) | 300/10 764 (3) |  |
| Diabetes |  |  |  | <0.01 |
| No | 25 279/26 058 (97) | 14 997/15 294 (98) | 10 282/10 764 (96) |  |
| Yes | 773/26 057 (3) | 297/15 294 (2) | 482/10 764 (5) |  |
| Living with HIV |  |  |  | <0.001 |
| No | 19 780/25 057 (79) | 11 507/15 098 (76) | 8 273/9 959 (83) |  |
| Yes | 5 277/25 057 (21) | 3 591/15 098 (24) | 1 686/9 959 (17) |  |
| Symptom duration |  |  |  | <0.001 |
| 0-4 days | 16 230/25 682 (63) | 9 035/14 887 (61) | 7 195/10 795 (67) |  |
| ≥5 days | 9 452/25 682 (37) | 5 852/14 887 (39) | 3 600/10 795 (33) |  |
| Hospital duration |  |  |  | 0.003 |
| <4 days | 10 565/25 999 (41) | 6 174/15 285 (40) | 4 391/10 714 (41) |  |
| ≥4days | 15.434/25 999 (59) | 9 111/15 285 (39) | 6 323/10 714 (59) |  |
| Oxygen therapy |  |  |  | <0.001 |
| No | 11 914/26 065 (46) | 8 495/15 281 (56) | 2419/10 784 (32) |  |
| Yes | 14 151/26 065 (54) | 6 786/15 281 (44) | 7 365/10 784 (68) |  |
| ICU admission |  |  |  | <0.001 |
| No | 25 668/26 062 (98) | 15 012/15 278 (98) | 10 656/10 784 (99) |  |
| Yes | 394/26 062 (2) | 266/15 278 (2) | 128/10 784 (1) |  |
| Outcome |  |  |  | <0.001 |
| No | 25 024/26 001 (96) | 14 818/15 273 (97) | 10 206/10 728 (95) |  |
| Died | 977/26 001 (4) | 455/ 15 273 (3) | 522/10 728 (5) |  |

ICU-intensive care unit
